# Consumption in Miners

**Published:** 1919-03-29

**Authors:** 


### Consumption in Miners.

MR. WALLACE THORNEYCROFT, Chairman of the Scottish Coal Users' Association, giving evidence before the Coal Commission, asserted that the reason of the low death-rate from phthisis among miners is that "the coal dust enters the lungs, but curiously enough its effect is beneficial, as it stimulates the lungs to throw off various deleterious matters." It is not impossible that the fine aseptic coal dust may act as a counter irritant in some instances, and may cause a salutary hyperæmia of the lungs, but in view of the disease anthracosis, which is caused by coal dust and which certainly predisposes to tuberculosis, we find it difficult to accept Mr. Thorneycroft's theory.

The low death-rate from phthisis among miners may be explained in many ways. Even though a pit may not be spacious, yet nowadays pits are well ventilated, probably better ventilated than the ordinary shop, ordinary workman's house, or ordinary factory. The work of mining, too, implies constant exercise of the muscles of the arms and chest, and such exercise is probably beneficial for the respiratory organs. The miner has better wages than most working-men, and therefore probably feeds better, and tuberculosis, as this war has amply demonstrated, is a disease of under-nutrition. Again, the miners are picked men of good physique and good muscular development. All these facts may help to explain the miner's comparative immunity from tuberculosis. And still another fact must be noticed. At one time tuberculosis was very rife among miners, and the death-rate from the disease was high. Now, mining is a hereditary occupation: it descends from father to son and remains in the same families for generations. Accordingly the miners of the present generation are survivors who have shown special resistance to the disease. It is a case of the survival of the fittest—the fittest to resist the tubercle bacillus. This selective process may have a good deal to do with the low death-rate from consumption among miners of the present day. It would be interesting in this connection to compare the death-rates in the older and newer mines, and the death-rate from consumption among the miners' womenfolk.

